# Hypothalamic Response to the Chemo-Signal Androstadienone in Gender Dysphoric Children and Adolescents

**DOI:** 10.3389/fendo.2014.00060

**Published:** 2014-05-28

**Authors:** Sarah M. Burke, Peggy T. Cohen-Kettenis, Dick J. Veltman, Daniel T. Klink, Julie Bakker

**Affiliations:** ^1^Center of Expertise on Gender Dysphoria, Neuroscience Campus Amsterdam, Department of Medical Psychology, VU University Medical Center, Amsterdam, Netherlands; ^2^Neuroendocrinology Group, Netherlands Institute for Neuroscience, Amsterdam, Netherlands; ^3^Department of Psychiatry, Neuroscience Campus Amsterdam, VU University Medical Center, Amsterdam, Netherlands; ^4^Center of Expertise on Gender Dysphoria, Department of Pediatric Endocrinology, VU University Medical Center, Amsterdam, Netherlands; ^5^GIGA Neuroscience, University of Liege, Liege, Belgium

**Keywords:** androstadienone, chemo-signal, fMRI, gender dysphoria, hypothalamus, puberty, sex difference

## Abstract

The odorous steroid androstadienone, a putative male chemo-signal, was previously reported to evoke sex differences in hypothalamic activation in adult heterosexual men and women. In order to investigate whether puberty modulated this sex difference in response to androstadienone, we measured the hypothalamic responsiveness to this chemo-signal in 39 pre-pubertal and 41 adolescent boys and girls by means of functional magnetic resonance imaging. We then investigated whether 36 pre-pubertal children and 38 adolescents diagnosed with gender dysphoria (GD; DSM-5) exhibited sex-atypical (in accordance with their experienced gender), rather than sex-typical (in accordance with their natal sex) hypothalamic activations during olfactory stimulation with androstadienone. We found that the sex difference in responsiveness to androstadienone was already present in pre-pubertal control children and thus likely developed during early perinatal development instead of during sexual maturation. Adolescent girls and boys with GD both responded remarkably like their experienced gender, thus sex-atypical. In contrast, pre-pubertal girls with GD showed neither a typically male nor female hypothalamic activation pattern and pre-pubertal boys with GD had hypothalamic activations in response to androstadienone that were similar to control boys, thus sex-typical. We present here a unique data set of boys and girls diagnosed with GD at two different developmental stages, showing that these children possess certain sex-atypical functional brain characteristics and may have undergone atypical sexual differentiation of the brain.

## Introduction

In humans, the odorous steroid 4,16-androstadien-3-one (androstadienone) has been studied intensively as a putative male modulator chemo-signal. Androstadienone, probably synthesized in the gonads (1), is secreted by the apocrine glands and can be found on the skin surface and axillary hairs (2), as well as in several body fluids including sweat and semen (3, 4). Higher concentrations of androstadienone in sweat have been found in men compared to women (5, 6). Exposure to androstadienone has been shown to affect women’s mental state (7–10), and to elicit physiological responses in a sex-dependent manner (7, 9, 11–15). Moreover, smelling androstadienone had an impact on women’s hormone levels (16) and affected their behavioral responses dependent on the phase of the menstrual cycle (17).

In line with the effects androstadienone had on the autonomic nervous system and on behavior, studies conducted by Savic et al. (18), using positron emission tomography (PET), showed that smelling androstadienone induced a response in the hypothalamus of heterosexual women but not in heterosexual men. The latter only showed an activation in brain areas belonging to the main olfactory system, such as the piriform cortex and amygdala (18). Thus, olfactory stimulation with androstadienone offers a relatively simple and objective experimental procedure for investigating functional sex differences in the human brain. Using functional magnetic resonance imaging (fMRI), we recently replicated the finding of women showing a stronger hypothalamic activation than men, after smelling the highest (10 mM) out of three concentrations tested (19).

Secretion of androstadienone increases substantially during puberty. As a result, sex-related changes in olfactory sensitivity to androstenes have been reported during adolescence (20–23) with male adolescents exhibiting more anosmia to androstadienone, and an increase of odor threshold with age, compared to female adolescents. It may be inferred that the decrease in sensitivity to odorous steroids in pubescent boys is related to their increased production of endogenous androgens during puberty, probably reflecting adaptation toward (male) body odors.

Sisk and colleagues (24, 25) proposed a two-stage model of sexual differentiation of brain and behavior, in which early perinatal organizational effects of steroid hormones are followed by a second steroid-dependent sensitive phase of neurodevelopment during adolescence. Thus, puberty may be considered an organizational period in itself, in which sex differences in brain morphology and function are established or consolidated. Therefore, our first aim was to investigate whether the hypothalamic response to androstadienone develops during puberty, as part of sexual maturation, or whether it would already be present in pre-pubertal children and thus established during early brain development. Thus, we tested whether the response to androstadienone varied as a function of sex and pubertal status in four groups of pre-pubertal and adolescent boys and girls.

Of note, further studies by Savic et al. (26–28) showed that hypothalamic activation upon exposure to androstadienone also depended on sexual orientation (27, 28) and gender identity. Adult men diagnosed with gender dysphoria (GD) [DSM-5, Ref. (29)], and thus having a female gender identity showed a sex-atypical hypothalamic response, which reflected more on their experienced gender than their natal sex (26). This is in line with the hypothesis that individuals, diagnosed with GD might have undergone a sex-atypical programing of the nervous system (30, 31). The etiology of GD is currently unknown, however, a multitude of factors, ranging from adverse psycho-dynamic parent–child interactions (32), an anxious personality predisposition (33), genetic risk factors (34), and exposure to atypical levels of perinatal sex steroids during a critical period of sexual differentiation of the brain (35) have all been proposed to facilitate the development of GD.

At the Center of Expertise on Gender Dysphoria in Amsterdam, the current treatment protocol allows adolescents diagnosed with GD that persisted from childhood into adolescence to start treatment with gonadotropin-releasing hormone analogs (GnRHa) from the age of 12 years, to suppress endogenous gonadal stimulation and thus the development of irreversible sex characteristics of the natal sex (36). From the age of 16 years on, as a first step in sex reassignment they receive cross-sex hormone treatment, i.e., biological boys receive estrogens and biological girls receive androgens (37, 38).

A second aim of the current study was therefore to explore whether children and adolescents, diagnosed with GD, would show brain responses that reflect their expressed/experienced gender rather than their natal sex, and whether these would vary as a function of their developmental phase. Four groups of subjects, all diagnosed with GD, participated in the current study: girls and boys that were pre-pubertal and treatment-naïve, and girls and boys that were adolescent in age (though in hypogonadal state due to GnRHa treatment). None of our participants received cross-sex hormones at the time of data acquisition.

## Materials and Methods

### Subjects

The initial study sample consisted of a total of 158 participants. Four subjects were excluded from further analysis, because of anatomical anomalies (one adolescent), technical errors during data collection (one child), or because the diagnosis GD had been revised since their participation in the study (two boys with GD in remission). All participants diagnosed with GD, were recruited via the Center of Expertise on Gender Dysphoria at the VU University Medical Center in Amsterdam. The control participants were recruited via several primary and secondary schools in the Netherlands and by inviting friends and relatives of the participants with GD.

The children sample consisted of 19 control girls [mean years of age (*M*) = 9.7, standard deviation (SD) = 0.9], 20 control boys (*M* = 9.5, SD = 1.1), 17 girls with GD (*M* = 9.6, SD = 1.1), and 19 boys with GD (*M* = 10.4, SD = 0.9). All children underwent a short physical examination by a pediatric endocrinologist (Daniel T. Klink) in order to ascertain their pre-pubertal status (Tanner stage 1) (39, 40).

The adolescent groups consisted of 21 control girls (*M* = 16.3, SD = 0.9), 20 control boys (*M* = 15.0, SD = 0.6), 21 girls with GD (*M* = 16.1, SD = 0.8), and 17 boys with GD (*M* = 15.3, SD = 1.2). The adolescent participants, diagnosed with GD, had been treated monthly with 3.75 mg of Triptorelin (Decapeptyl-CR^®^, Ferring, Hoofddorp, the Netherlands) by injection for on average 24 months (range 2-48 months), resulting in complete suppression of gonadal hormone production. Female adolescent controls were tested randomly according to their menstrual cycle and 11 out of 21 control girls reported using hormonal contraception.

### Assessments and subject characteristic

Sexual orientation was difficult to assess, especially in the pre-pubertal sample, because most children were simply too young to be able to report their sexual orientation. Therefore, current or presumed future sexual attraction was assessed by asking whether the participant had ever been in love with somebody, and if yes, whether that person was a boy or a girl. Normal olfactory function was ascertained by means of an extended version of the “Sniffin’ Sticks” test battery (32-item odor identification test and olfactory threshold measurement) (41–43). Furthermore, participants were asked to report the perceived intensity of a 10 mM androstadienone solution (on a scale from 0 to 10). Separately, for the adolescent and the children samples, one-way analyses of variance (ANOVA) were conducted, using the Statistical Package for the Social Sciences, version 20 (SPSS Inc., Chicago, IL, USA), testing whether the four gender groups differed on any of these measures. Bonferroni correction for multiple comparisons was applied for *post hoc* tests, considering a threshold of *p* < 0.05 as statistically significant. All subjects and their legal guardians gave their informed consent according to the Declaration of Helsinki, and the study was approved by the Ethics Committee of the VU University Medical Center Amsterdam (application number NL31283.029.10).

### Olfactory stimulation

Androstadienone (Steraloids Inc., Newport, RI 02840, USA) was diluted in propylene glycol (Sigma) to a concentration of 10 mM, according to the “high” concentration used in our previous study (19). The volume of the solution used during the fMRI experiments was 20 ml. Olfactory stimuli were delivered through a tubing system to the subjects’ nostrils by means of a custom-built air-dilution olfactometer [for details of the olfactometer set-up and procedure see Ref. (19)]. With a total air flow of about 1 L/min, during “ON” periods every 2 s, the odor was delivered during 1 s, while during “OFF” periods subjects received odorless air.

### Image acquisition

Scans were performed on a 3.0-T GE Signa HDxt scanner (General Electric, Milwaukee, WI, USA). A gradient echo, echo planar imaging sequence was used for functional imaging (19.2 cm^2^ field of view, TR of 1950 ms, TE of 25 ms, an 80° flip angle, isotropic voxels of 3 mm, and 36 slices). Before each imaging session, a local high-order shimming technique was used to reduce susceptibility artifacts. A scanning session consisted of six alternating ON-OFF cycles over 108 volumes in a classical block design (one block consisted of nine volumes), lasting 3.6 min. For co-registration with the functional images, a T1-weighted scan was obtained (3D FSPGR sequence, 25 cm^2^ field of view, TR of 7.8 ms, TE of 3.0 ms; slice thickness of 1 mm, and 176 slices).

### Image processing

Data analysis was performed with SPM8 software (Statistical Parametric Mapping; Wellcome Department of Imaging Neuroscience, Institute of Neurology at the University College London, UK) implemented in Matlab R2009b (Math Works Inc., Natick, MA, USA). Functional images were slice-timed and realigned to the mean image, followed by unwarp. Applying the “New Segment” and “Create Template” options of the DARTEL (Diffeomorphic Anatomical Registration Through Exponentiated Lie Algebra) toolbox, structural images were segmented. Then, gray matter and white matter images were used for creating age-group specific templates (one for the children and the adolescents sample each), registered in Montreal neurological institute (MNI) space. Functional images were spatially normalized to their respective group-template, applying each individual’s DARTEL flow field, and finally, images were smoothed by means of a 5-mm full width half maximum (FWHM) isotropic Gaussian kernel.

Individual image data were analyzed using boxcar regressors convolved with a synthetic hemodynamic response function and a first-order time-modulation (TM) regressor to test for possible effects of adaptation/sensitization to androstadienone. In order to account for assumed late “wash-out” effects during OFF blocks and an early peak response to the odor stimulation during ON blocks, first-level contrast images were built by subtracting the second half (b) of the OFF blocks (four volumes) from the first part (a) of the ON blocks (four volumes). Accordingly, this was done with the associated TM regressor blocks. Further, based on the image realignment process, individual head jerks were identified (>1 mm displacement) (44). Together with the six motion parameters, these so-called scan nulling regressors were included in every first-level design matrix to account for the effects of excessive head motion.

### Statistical analyses

First, in order to test whether the sex difference in response to androstadienone was present in both developmental control groups, and whether that sex difference in responsiveness varied as a function of adaptation/sensitization to the odor, we conducted a *sex* (control boys, control girls) by *odor stimulation* ANOVA, for both the pre-pubertal and the adolescent control groups. The factor *odor stimulation* consisted of two levels, a regressor modeling the condition ON_a_-OFF_b_ effect, thus the hypothesized hypothalamic response to the odor, and a first-order parametric modulation TM ON_a_-TM OFF_b_ regressor, which signifies how well the hypothalamic response correlates with changes over time, thus modeling possible effects of adaptation or sensitization.

Second, by means of four *gender* by *odor stimulation* ANOVAs separately for the pre-pubertal and the adolescent groups, we tested whether boys and girls diagnosed with GD differed significantly in response to the steroid odor in comparison to their respective natal sex or their experienced gender control group.

Analyses were restricted to the hypothalamus area as region of interest (ROI), defined [with Marsbar (45)] as a sphere (centered at MNI coordinates *x* = 0, *y* = -10, *z* = -7; with a 7-mm radius), and based on anatomical demarcations following (46) and (47). The threshold for statistical significance was set at *p* < 0.05 family-wise error (FWE)-corrected for the extent of the hypothalamus ROI.

## Results

### Psychophysics and subject characteristics

Demographic, self-report, and subject characteristics are presented in Table 1. The adolescent boys with GD were significantly younger and less physically mature than the three other adolescent groups. The pre-pubertal boys with GD were significantly older than the pre-pubertal control boys. Therefore, in all group comparisons involving boys with GD, age was included as covariate. As expected, both adolescent control groups differed significantly from the boys and girls diagnosed with GD with respect to reported sexual orientation. There were no group differences in olfactory performance and on ratings of the perceived intensity of androstadienone.

**Table 1:** **Subject characteristics and psychophysiological data**.

			Pre-pubertal children						Adolescents					
			Ctrl girls	Ctrl boys	Girls with GD	Boys with GD	*F*(df)	*p*-Value	Ctrl girls	Ctrl boys	Girls with GD	Boys with GD	*F*(df)	*p*-Value
Group size	*N*		19	20	17	19			21	20	21	17		
Age in years	Mean (SD)		9.7 (0.9)	9.5 (1.1)	9.6 (1.1)	10.4 (0.9)	3.1 (3.71)	**0.033**	16.3 (0.9)	15.9 (0.6)	16.1 (0.8)	15.3 (1.2)	4.6 (3.75)	**0.005**
Pubertal stage	Mean (SD)	P	1	1	1	1	–	–	4.2 (0.7)	4.7 (0.7)	4.7 (0.6)	3.1 (1.1)	17.4 (3.74)	**<0.001**
		G/M^a^	1	1	1	1	–	–	4.1 (0.8)	4.1 (0.8)	4.1 (1.1)	3.1 (0.8)	5.1 (3.74)	**0.003**
Sexual orientation	% (*N*)	Gynephilic	–	66.0 (13)	17.6 (5)	21.1 (4)			–	100 (20)	100 (21)	–		
		Androphilic	89.5 (17)	–	29.4 (3)	42.1 (8)			100 (21)	–	–	70.6 (12)		
		Ambiphilic	–	–	5.9 (1)	–	1.1 (3.71)	0.364	–	–	–	5.9 (1)	33.3 (3.74)	**<0.001**
		Don’t know	10.5 (2)	35.0 (7)	47.1 (8)	36.8 (7)			–	–	–	17.6 (3)		
		Missing	–	–	–	–			–	–	–	5.9 (1)		
Androstadienone^b^	Mean (SD)		6.1 (2.4)	6.9 (1.5)	6.2 (2.7)	5.7 (2.6)	0.7 (3.55)	0.548	5.7 (2.3)	4.8 (1.9)	5.5 (1.9)	4.6 (2.0)	1.1 (3.63)	0.351
Sniffin’ sticks	Mean (SD)	Threshold	7.3 (3.9)	6.2 (4.1)	8.5 (3.4)	8.6 (3.4)	1.6 (3.58)	0.212	9.9 (3.3)	8.5 (3.4)	8.1 (2.9)	9.1 (3.4)	1.1 (3.68)	0.356
		Identification	21.5 (3.6)	18.5 (3.8)	19.1 (4.0)	19.9 (5.3)	1.8 (3.66)	0.162	25.2 (3.8)	23.9 (2.8)	23.5 (3.9)	23.7 (2.8)	1.0 (3.71)	0.419

*Ctrl, control; GD, gender dysphoria; *N*, group size; SD, standard deviation; P, pubic hair growth; G, genital development; M, breast development*.

*^a^G applies for natal boys and M for natal girls; pubertal stages were assessed by means of the five-point (1 = pre-pubertal, 5 = post-pubertal) Tanner Maturation Scale*.

*^b^Androstadienone was rated for perceived intensity on a scale from 0 to 10; scores on the Sniffin’ sticks threshold test range from 1 (low sensitivity) to 16 (high sensitivity); scores on the odor identification test range from 1 to 32. Bold font indicates statistically significant effects*.

### Sex differences in hypothalamic activation – effects of puberty

In order to determine whether hypothalamic activation upon smelling androstadienone is dependent on puberty, we conducted separate ANOVAs in the pre-pubertal and the adolescent groups, and compared hypothalamic activation in control girls to that of control boys during exposure to androstadienone. Results are displayed in Table 2.

**Table 2:** **ANOVA results showing sex differences in hypothalamus activations in the control groups, and *sex-atypical* hypothalamic activations, reflecting their experienced gender, in individuals with GD**.

	Effect	*Z*_max_	*x*	*y*	*z*	*N*	*P*_FWE_
**CTRL GIRLS > CTRL BOYS**							
Children	Gender	2.5	-6	-12	-5	30	0.202
	Odor	2.4	-2	-15	-2	39	0.244
	ON-OFF	1.8	0	-10	-14	1	0.456
	ON_TM_-OFF_TM_	3.2	-6	-12	-5	92	**0.033**
		2.9	6	-7	-8		
Adolescents	Gender	1.7	-3	-7	-2	17	0.091
	Odor	1.7	6	-7	-6	8	0.655
	ON-OFF	3.4	6	-7	-8	1	0.522
	ON_TM_-OFF_TM_	2.5	-6	-12	-5	115	**0.019**
**CTRL GIRLS > GIRLS WITH GD**							
Children	No effects				
Adolescents	Gender	3.5	6	-7	-9	76	**0.016**
	Odor	1.7	-6	-7	-6	1	0.627
	ON-OFF	–	–	–	–	–	–
	ON_TM_-OFF_TM_	4.1	6	-7	-9	214	**0.002**
**BOYS WITH GD > CTRL BOYS**							
Children	Gender	1.8	2	-14	-2	4	0.447
	Odor	1.9	0	-10	0	3	0.436
	ON-OFF	1.7	-4	-6	-4	12	0.437
	ON_TM_-OFF_TM_	1.9	0	-10	-12	31	0.329
		2.4	0	-14	-12	
Adolescents	Gender	2.2	6	-8	-6	11	0.237
	Odor	3.0	0	-14	-12	1	0.341
	ON-OFF	1.8	2	-14	-2	36	**0.046**
	ON_TM_-OFF_TM_	–	–	–	–	–	–
**BOYS WITH GD > GIRLS WITH GD**							
Children	No effects				
Adolescents	Gender	–	–	–	–	–	–
	Odor	1.9	3	-12	-9	12	0.535
	ON-OFF	–	–	–	–	–	–
	ON_TM_-OFF_TM_	2.1	3	-10	-2	26	0.323

*Ctrl, control; GD, gender dysphoria; Z_max_, *z*-value of peak activation voxel; *x y z*, coordinates in Montreal Neurological Institute (MNI) space; *N*, number of clustering voxels (cluster size); FEW, family-wise error; the effects “Gender” and “Odor” indicate omnibus F-contrasts for the factors *gender* and *odor stimulation*; “ON-OFF” and “ON_TM_-OFF_TM_” indicate directional *t*-contrasts for the condition effect and the first-order time-modulation regressor, respectively. Bold font indicates statistically significant effects*.

**Figure 1:**
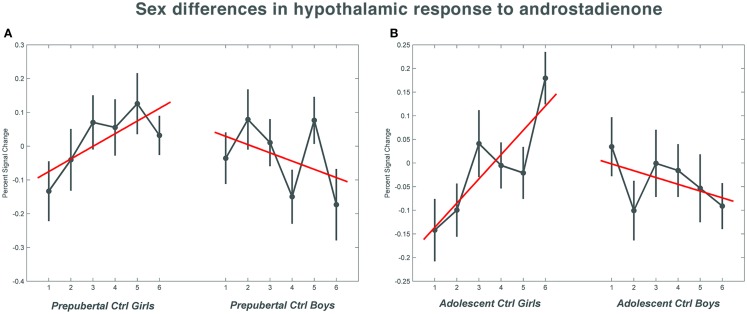
**The mean percent signal change for the hypothalamic region of interest during the course of the scanning session in the control groups**. **(A)** Shows the responses in the pre-pubertal boys and girls; **(B)** shows the changes in activation over time in the adolescent control groups. Numbers 1–6 indicate the six time bins of the odor stimulation block design. Irrespective of pubertal status, both female age groups showed a significant increase (*p* = 0.033 in the pre-pubertal girls; *p* = 0.019 in the adolescent girls) in activation with repeated exposure to androstadienone, whereas hypothalamic activations tended to decrease in boys toward the end of the odor stimulation. The data for this figure was visualized using the toolbox rfxplot by Gläscher (55).

**Figure 2:**
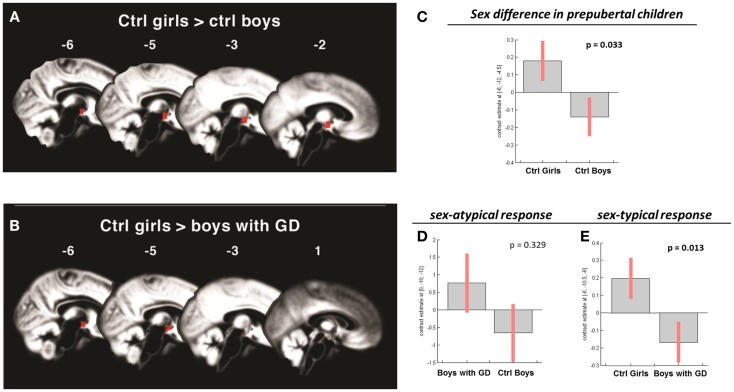
**(A,B)** Show the anatomic locations of the significant hypothalamic activations, indicated in red, in response to the chemo-signal androstadienone in the pre-pubertal age groups. The numbers above each sagittal plane represent the *x*-axis coordinates in Montreal Neurological Institute space. All voxels within the hypothalamic region of interest, surviving the statistical threshold of *p* < 0.05 (FWE-corrected) are shown. **(C–E)** Show the bar graphs of the corresponding group contrasts for the first-order time-modulation regressor, indicating group differences in sensitization to the steroid odor. **(C)** Displays the sex difference, i.e., between control girls and boys; **(D)** shows the non-significant sex-atypical (i.e., female-like) and **(E)** indicates the significant sex-typical (i.e., male-like) response of boys with gender dysphoria.

Pre-pubertal girls and boys did not differ in terms of general hypothalamic responsiveness (condition effect ON > OFF) to androstadienone, but directional *t*-tests showed that the sex difference (girls > boys) was significantly modulated by the effect of the TM regressor (*t* = 3.3; *p* = 0.033), indicating that hypothalamic activation in boys differed from that of the girls during the course of the scanning session, thus suggesting differences in adaptation and/or sensitization to the odor between groups (see Figures 1A and 2A,C).

**Figure 3:**
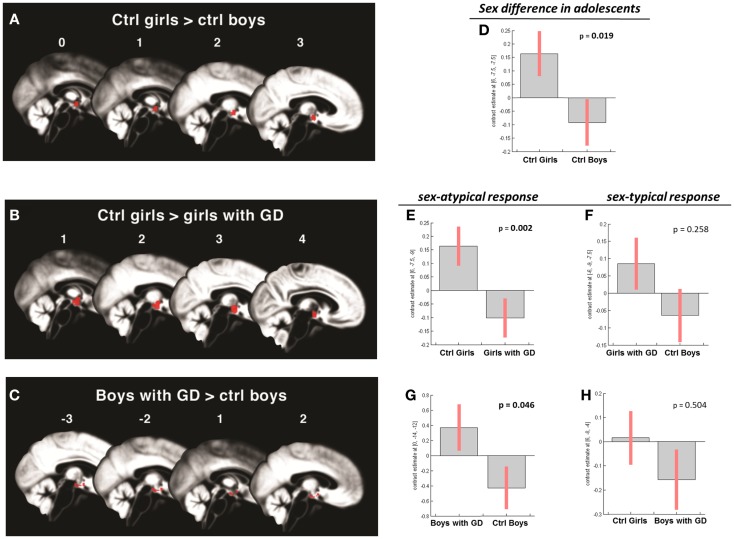
**(A–C)** Show the anatomic locations of the significant hypothalamic activations, indicated in red, in response to the chemo-signal androstadienone in the adolescent age groups. The numbers above each sagittal plane represent the *x*-axis coordinates in Montreal Neurological Institute space. All voxels within the hypothalamic region of interest, surviving the statistical threshold of *p* < 0.05 (FWE-corrected) are shown. **(D–H)** Show the bar graphs of the corresponding group contrasts. **(D–E)** Show the significant group contrasts of the first-order time-modulation regressor, indicating sensitization to the steroid odor in control girls compared with **(A)** control boys and **(E)** girls with gender dysphoria. **(F)** Shows the non-significant sex-typical (female-like) response of girls with gender dysphoria. **(G)** Displays the significant female-typical response of the boys with gender dysphoria for the contrast of the condition ON > OFF, irrespective of any effects of time; **(H)** shows the non-significant male-typical response of boys with gender dysphoria.

Accordingly, similar comparisons were done in the adolescent groups. Again, the sex difference in hypothalamic activation (girls > boys) was significantly dependent on the factor time (*t* = 3.5; *p* = 0.019) (see Figures 1B and 3A,D). Visual inspection of the data (see Figure 1) revealed that control girls’ activation increased with repeated exposure to androstadienone (sensitization), particularly toward the end of the session, whereas control boys’ responsiveness to the steroid odor seemed relatively stable, showing a slight decrease in activation during the course of the stimulation session.

### Gender identity and hypothalamic activation

In order to test whether individuals diagnosed with GD would show a hypothalamic activation that reflected their experienced gender, and whether this brain response would be different for pre-pubertal and adolescent subjects, four ANOVAs were conducted, in which control girls were compared to girls with GD, and control boys were compared to boys with GD (see Table 2). In addition, we investigated the reverse effects, i.e., whether individuals with GD showed sex-typical activations, and thus deviated from their experienced gender by comparing girls with GD to control boys, and boys with GD to control girls (see Table 3). Comparing girls with GD to boys with GD revealed no significant differences, thus the sex difference in response to androstadienone observed in the control groups was absent in the groups diagnosed with GD.

**Table 3:** **ANOVA results showing *sex-typical* hypothalamic activations, reflecting their natal sex, in individuals with GD**.

	Effect	*Z*_max_	*x*	*y*	*z*	*N*	*P*_FWE_
**GIRLS WITH GD > CTRL BOYS**							
Children	No effects				
Adolescents	Gender	–	–	–	–	–	–
	Odor	2.5	6	-9	-5	25	0.203
	ON-OFF	1.7	-6	-7	-5	2	0.512
	ON_TM_-OFF_TM_	2.3	-6	-9	-8	25	0.258
**CTRL GIRLS > BOYS WITH GD**							
Children	Gender	3.0	-6	-10	-6	35	0.065
	Odor	2.6	3	-16	-9	27	0.168
	ON-OFF	1.6	-2	-16	-6	4	0.550
	ON_TM_-OFF_TM_	3.5	-6	-10	-6	126	**0.013**
Adolescents	Gender	2.2	-2	-4	-10	5	0.336
	Odor	1.5	2	-14	-12	4	0.699
	ON-OFF	–	–	–	–	–	–
	ON_TM_-OFF_TM_	1.7	6	-8	-4	7	0.504
		1.6	-6	-12	-8	16	0.553
**GIRLS WITH GD > BOYS WITH GD**							
Children	No effects				
Adolescents	Gender	–	–	–	–	–	–
	Odor	1.9	3	-12	-9	12	0.535
	ON-OFF	–	–	–	–	–	–
	ON_TM_-OFF_TM_	1.9	-6	-7	-11	1	0.459

*Ctrl, control; GD, gender dysphoria; Z_max_, *z*-value of peak activation voxel; *x y z*, coordinates in Montreal Neurological Institute (MNI) space; *N*, number of clustering voxels (cluster size); FWE, family-wise error; the effects “Gender” and “Odor” indicate omnibus F-contrasts for the factors *gender* and *odor stimulation*; “ON-OFF” and “ON_TM_-OFF_TM_” indicate directional *t*-contrasts for the condition effect and the first-order time-modulation regressor, respectively. Bold font indicates statistically significant effects*.

#### Girls diagnosed with GD

None of the comparisons between pre-pubertal control girls and pre-pubertal girls with GD revealed any differences in hypothalamic activation upon smelling androstadienone. However, no sex-typical effects (i.e., a female-typical hypothalamus response), when compared to control boys, could be confirmed. Thus, pre-pubertal girls with GD neither differed significantly from their experienced (control boys) nor from their natal sex (control girls) in terms of hypothalamic activation when smelling androstadienone.

In contrast, the comparison of adolescent control girls to girls diagnosed with GD revealed a significant effect of *gender* (control girls > girls with GD), which was mainly explained by the effect of the TM regressor (*t* = 4.3; *p* = 0.002) (see Table 2; Figures 3B,E). Thus, control girls showed a significantly stronger hypothalamic response to androstadienone over time as described earlier, whereas the activation in girls with GD, similar to adolescent control boys, remained stable throughout the scanning session. The reverse group comparisons (girls with GD > control boys) revealed no significant effects (see Figure 3F), indicating that adolescent girls with GD showed no hypothalamic activation upon smelling androstadienone as was observed in adolescent control boys.

#### Boys diagnosed with GD

No significant effects were revealed when comparing pre-pubertal boys with GD with control boys. In contrast, a significant effect of *gender*, driven by the TM regressor (*t* = 3.5; *p* = 0.013 FWE-corrected) was revealed when comparing pre-pubertal boys with GD to control girls, indicating that pre-pubertal boys with GD showed a pattern of hypothalamic activation that was similar to that of the pre-pubertal control boys (see Table 3; Figures 2B,D,E).

When adolescent boys with GD were compared to adolescent control boys we observed a significant effect of condition (ON > OFF) (*t* = 1.8; *p* = 0.046) (see Table 2; Figures 3C,G,H). Thus, adolescent boys with GD showed a significantly stronger, thus sex-atypical response to androstadienone compared to control boys, irrespective of the factor time. The reverse contrast (control girls > adolescent boys with GD), i.e., testing whether adolescent boys with GD would show an activation according to their natal sex, revealed no significant activations. Thus, adolescent boys with GD showed female-typical hypothalamic responses upon smelling androstadienone without, however, any effects due to sensitization.

## Discussion

### Sex differences in hypothalamic activation – effects of puberty

The present study is, to our knowledge, the first to demonstrate that sex differences in hypothalamic activation upon smelling androstadienone are already present before puberty, and thus may be considered as a sex difference established during early brain development. We found that pre-pubertal as well as adolescent control girls showed a stronger hypothalamic activation compared to boys (at both developmental stages), and that this sex difference was crucially modulated by effects of sensitization to androstadienone.

Previous psychophysiological studies in children and adolescents have shown that olfactory sensitivity to androgenic odors differed between boys and girls during development, due to the increased production of endogenous androgens by boys during puberty and the assumed resulting adaptation to their own body odors (20–23). These findings suggested that puberty plays an important role in the development of sex differences in the olfactory sensitivity to androstadienone (48). Therefore, we hypothesized that the hypothalamic response in males, in contrast to that in females, would similarly be subject to neuronal adaptation after repeated exposure to the steroid odor. However, our results suggested only slight neuronal adaptation effects in boys, their activation remained relatively stable throughout the scanning session; instead, we found that girls showed an increase in hypothalamic responsiveness during the course of the odor stimulation. In line with the idea that androstadienone may function as a male modulator chemo-signal, our findings thus suggest that androstadienone indeed affects brain functions in females. Moreover, we showed that the sex difference in hypothalamic activation was not related to any hormonal or sexual maturation-related changes during puberty. Therefore, sex differences in neural responsiveness are probably not associated with differences in olfactory sensitivity to androstadienone and thus, its subjectively perceived intensity. Accordingly, we did not find any significant sex differences in the reported intensity ratings of androstadienone in both the pre-pubertal and adolescent groups.

### Gender identity and hypothalamic activation

Gender dysphoria has been hypothesized to develop due to an altered sexual differentiation of the body and the brain during early development (30, 31). Here, we investigated a unique data set of individuals with GD at two different developmental stages, in order to determine whether they would respond to androstadienone in accordance with their natal sex, rather than their experienced gender. We found that both, adolescent girls and boys with GD showed hypothalamic activations that reflected their experienced gender. The sex difference that we observed in the control groups, was absent in both age groups of boys and girls diagnosed with GD. However, pre-pubertal girls with GD showed no differences in hypothalamic activation compared to both control boys and girls, whereas pre-pubertal boys with GD showed significant sex-typical hypothalamic activations and thus, in accordance with their natal sex. These findings thus suggest that individuals with GD possess certain functional brain characteristics, i.e., hypothalamic activation to androstadienone, of their experienced gender and thus that they may have undergone atypical neuronal sexual differentiation. However, this can only be observed reliably in adolescents with GD.

#### Girls diagnosed with GD

Pre-pubertal girls with GD did not differ in hypothalamic activation from control girls indicating that they did not show a male-typical response. However, they also did not show a female-typical hypothalamic activation, when compared to control boys, indicating that they did not differ from either of the control groups. It is possible that pre-pubertal girls with GD constitute a rather heterogeneous group with respect to future persisting GD. It has been shown that only about 15.8% of the childhood GD cases will eventually lead to adult GD (49). However, women present more often early-onset cases (50) and girls have overall higher persistency rates of GD into adolescence compared to boys (51). Thus, our finding that pre-pubertal girls with GD did not show a clear-cut female- or male-typical response to androstadienone awaits further confirmation in the future, when it will be known who of our participants showed persisting GD into adolescence and adulthood.

The comparison of adolescent female controls versus adolescent girls with GD revealed very similar results as the control group comparisons, i.e., stronger hypothalamic activations in those subjects experiencing a female gender identity, and this effect was mainly driven by effects over time, reflecting sensitization. Thus, adolescent girls with GD responded remarkably like their experienced gender (control boys). While speculative, these findings fit with the idea that this group of girls with GD (who are more homogenous in terms of future persisting GD compared to the pre-pubertal groups) may have had a more male-typical perinatal hormonal environment, resulting in the development of certain typically male functional sex characteristics of the brain. The hypogonadal state of adolescent girls with GD using GnRHa at the moment of data acquisition is not likely to account for these findings, since our results in the pre-pubertal control groups suggest that hypothalamic responsiveness to androstadienone is probably independent of puberty, and thus not affected by circulating sex hormones. Furthermore, animal studies showed that sexually dimorphic responses to volatile urinary odors were not dependent on circulating sex steroids, but rather developed under the influence of organizational effects of sex hormones (52, 53).

It should be noted, though, that sexual orientation of the participants with GD might present a confounding factor. Berglund et al. (27) showed that hypothalamic responsiveness to androstadienone in lesbian women was comparable to that of heterosexual men. In the literature, the majority of natal females with GD are reported to be gynephilic (50, 54), which was true as well for our group of adolescent gender dysphoric girls. Therefore, it cannot be ruled out that the resemblance in hypothalamic activation with the control boys might be due to their shared sexual orientation rather than their shared gender identity.

#### Boys diagnosed with GD

The hypothalamic response to androstadienone in the pre-pubertal boys with GD was not significantly stronger than that shown by the pre-pubertal control boys. Moreover, the reverse contrast (control girls > boys with GD) revealed that pre-pubertal control girls showed significantly stronger hypothalamic activation, implying that boys with GD responded according to their natal sex. Again, it is likely that the younger, pre-pubertal sample of boys with GD constitutes a rather heterogeneous group in terms of future persistence of their gender dysphoric feelings (49, 51). Therefore, they may show more variability in hypothalamic responsiveness to androstadienone.

Adolescent boys with GD showed significantly stronger, thus sex-atypical hypothalamic activations, compared to control boys, although this effect was not modulated by any effects of time, thus sensitization to androstadienone, as we did observe in the control girls. Accordingly, the reverse comparison (control girls > boys with GD) revealed no significant effects. The female-typical activation in adolescent boys with GD is in line with a previous study by Berglund et al. (26), who reported that adult men with GD differed significantly from a group of male controls in terms of hypothalamic activation during exposure to androstadienone. Their adult participants with GD were matched to control men with regard to hormonal status and gynephilic sexual orientation, whereas in our study adolescent boys with GD received GnRHa at the moment of data acquisition and the majority of our participants reported to have an androphilic sexual orientation. Thus, despite differences with regard to the participants’ age, hormonal status, and sexual orientation, the findings of both studies suggest that males with GD possess certain female-typical brain functions, and may therefore have undergone sex-atypical early brain development.

Some limitations of the present study should be mentioned and may be addressed in future research. In most comparisons, the gender differences in hypothalamic response to androstadienone showed relatively small effect sizes. These effect sizes, in combination with relatively small group sizes (especially that of the pre-pubertal girls with GD with *N* = 17), suggest that a lack of statistical power may be related to our failure in finding any significant effects for particularly this group. Therefore, additional analyses in other functional and structural (e.g., gray and white matter volumes or diffusion tensor imaging data) MRI measures, which are in preparation, should corroborate the present preliminary findings. Sexual orientation was difficult to assess in our young groups of participants. We therefore estimated their (future) sexual orientation by asking whether he/she had ever been in love. However, any relationship between sexual orientation and the response to androstadienone could not reliably be investigated in our groups.

In summary, the present study is the first to demonstrate that sex differences in hypothalamic activation upon smelling the chemo-signal androstadienone are not acquired during sexual maturation, under the influence of gonadal hormones during puberty, but may be considered hard-wired responses, which already can be observed in pre-pubertal children. Moreover, the current study is the first to explore sex-atypical hypothalamic responses to androstadienone in male and female individuals with GD at two different developmental stages. Our results indeed suggest that individuals with GD possess certain functional brain characteristics of their experienced gender and may have undergone atypical neuronal sexual differentiation.

## Author Contributions

Julie Bakker and Sarah M. Burke designed the study set-up. Sarah M. Burke performed the neuroimaging experiments, conducted the data analyses with advice of Dick J. Veltman, and wrote the manuscript. Daniel T. Klink performed the clinical assessments. Peggy T. Cohen-Kettenis, Dick J. Veltman, and Julie Bakker supervised the project. All authors contributed to interpretation of the data and revisions of the manuscript.

## Conflict of Interest Statement

The authors declare that the research was conducted in the absence of any commercial or financial relationships that could be construed as a potential conflict of interest.
